# Transactions of the Pathological Society of Philadelphia

**Published:** 1840-02-01

**Authors:** 


### TRANSACTIONS OF THE PATHOLOGICAL SOCIETY OF PHILADELPHIA.

*January 6th*, 1840.

The President, Dr. GERHARD, in the Chair.

*Hypertrophy of the Heart—Aneurism of the Aorta—repeated attacks of Apoplexy—Pulmonary Apoplexy, &c.—Death—Autopsy.* By C. W. PENNOCK, M. D.

E. Newport, a black woman, aged 50, was brought into the wards of the Philadelphia Hospital on the 18th of October, 1839, with hemiplegia. The right leg and arm were perfectly powerless, but without rigidity; sensation on the affected side greatly impaired, patient evincing pain only when pinching of its surface was very forcible; respiration slow, but unaccompanied by stertor; distortion of the face, the mouth drawn towards the left; apparently conscious of surrounding objects; voice unintelligible.

The treatment instituted consisted in free depletion by venesection, cupping the head, insertion of a seton in the nucha, exhibition of the active cathartics, (infusion of senna and sulph. magnesia.) Diet restricted to farinaceous articles. In the course of two weeks, a manifest melioration of symptoms took place; the voice became more distinct; memory somewhat improved; some intelligence; motion, which was first manifested in the great toe, was partially restored to the inferior extremity. At this time, Dr. Baker, the resident physician, ascertained from her, that her health had been good until four years before, at which time her catemenia suddenly ceased; she then became subject to cephalalgia, vertigo, and soon after, whilst walking in the street, fell, remaining without consciousness or motion for several hours, but recovered in a day, after free bleeding.

The attack which caused the hemiplegia, took place two weeks before her entrance into the hospital; it was preceded by pain in the head, vertigo, dimness of sight, and increased palpitations of the heart.

On the 7th of November, the patient presented the following symptoms: Expression of countenance dull; stupid; cheeks puffy, lips tumid, drawn towards the left side of the face, whilst the tongue is protruded slightly towards the right; voice very thick, pronunciation of words difficult. Right arm almost powerless, not rigid, falls as an inert mass when elevated; shoulder can be very slightly raised by deltoid muscle. In the right leg and thigh motion is partially restored, the leg can be flexed on the thigh, and this towards the pelvis. Sensation is restored to both limbs. Intelligence very dull and limited. *Digestive apparatus.*—Tongue moist, covered by thick, white fur; appetite moderate, no thirst; three alvine evacuations daily; abdomen resonant, soft, not distended, and not painful on pressure. Liver extends from the fifth rib to an inch below the lower edge of right ribs; right hypochondrium distended more than the left.

*Chest.*—Marked prominence over the cartilages of right ribs and upper two-thirds of sternum, which are very salient; the space over the region of heart on left side also prominent. Ribs of right side almost immoveable during inspiration, whilst left chest is expanded. Percussion resonant immediately beneath acromial half of right clavicle; flat under sternal half, and beneath cartilages of right ribs; obscure, lateral and inferiorly. Posteriorly, the percussion is resonant above, obscure below. The left chest is resonant in space of two inches beneath the clavicle, and in the axilla; posterior percussion obscure in middle third, resonant elsewhere. Respiration over right side generally feeble, heard upon forced inspiration; it is bronchial near the sternum and over the posterior inferior third, accompanied by crepitant rhonchus. Respiration of left chest where it is resonant, is expansive, vesicular; dyspnœa; respiration thirty per minute.

*Heart.*—Marked circumscribed prominence over the region of the heart between the third and fifth rib, extending laterally from the sternum to the left nipple. The heart, measured by percussion, extends beyond the right margin of the sternum, nearly to the union of the cartilages with the ribs; lateral extent of its dulness on percussion beneath the second cartilage towards left, is four inches; under the third, five inches; under the fourth rib, six and a half inches, being one inch to left of the left nipple; this dulness extends over a space of which the outline coincides in shape with that of the pericardium. Perfect flatness on percussion exists in the præcordial space from the third rib downwards the length of the sternum; and laterally on the fourth rib, from the right edge of the sternum towards the left, four inches.

No respiration is heard in the præcordial space where the percussion is flat. Impulse of heart is very forcible in a space of two inches square near left nipple, and is followed by "the receding or back stroke" of Hope. Two sounds of the heart are heard in the region of the nipple—the first is a prolonged, rough, bellows sound, the second sound is exceedingly feeble, and is often masked by the roughness of the first.

Ausculting on the line of the fourth rib, towards the sternum, the first sound becomes shorter, clearer, and the second more marked; to the right of the sternum the second sound is nearly normal. Over the aortic valves, (sternum opposite the third ribs,) the first sound is rough, somewhat rasping; bellows sound in the second. The rasping sound is very intense immediately beneath the sternal half of right clavicle, at which point no second sound is heard; the impulse in this space is stronger than at the apex of the heart. Slight bellows sound accompanies the sounds of the heart as heard over the valves of the pulmonary artery, (under cartilage of the second rib near the left side of the sternum.) Beneath the cartilage of the third rib, one inch and a half to the left of the sternum, (mitral valve,) first sound is more whizzing than it is over the aortic valves. Carotid arteries enlarged, beat with great violence, accompanied by a marked *bruit de soufflet;* pulse at the wrist very strong, incompressible, regular, sixty per minute; right radial artery of much greater volume than the left.

(*Treatment.*—Frequent blood letting, general and topical; administration of digitalis; purging by infusion of senna and sulphate of magnesia, every other day. Diet, oatmeal gruel.)

The symptoms continued with but little change until the 2d December, when the pulse at the wrist was observed to be much diminished in volume; this alteration was most marked in the artery of the right arm, which, instead of being, as previously, of the size of a rattan, did not appear larger than a crow quill; pulse still remained corded. Patient was auscultated almost daily; and the cardiac sounds were observed to vary in character over the left portion of the præcordial region, from a slight bellows to a deep rasping sound; this change would often come on during the examination. On one occasion it was markedly sibilant—no corresponding change was manifested over the aortic valves.

On sixth of December, profuse salivation ensued after exhibition of five grains of calomel, in divided portions of a grain each hour; patient complained much of the pain of the tongue, which was much enlarged. Next day, same complaint, disposition more irrascible. On the 9th, tendency to stupor manifested, but aroused by questions; complains of no pain but of that of the mouth and tongue; deviation of the mouth towards the left, increased; decubitus on the right side. On the morning of the 10th, the day of her death, it was noted, that she was insensible to surrounding objects, except when roused by repeated questions; unable to protrude the tongue, which is red and swollen and dry; exudation of blood on mucous membrane of lips; right leg and arm perfectly motionless, no rigidity; sensation not impaired. *Chest.*—The anterior prominence beneath the sternal half of the right clavicle and the cartilages of the ribs, much increased, being most marked beneath the three upper ribs; the bulging of the præcordial region also greater. Dulness on percussion of the region of the heart observed over a greater space than at first note; beneath second rib, obscurity extends five inches; on a line with fourth rib seven inches; and beneath the nipple, to the cartilage of sixth rib. Impulse of the heart extends over a large space—it is strong at, and below the nipple: it is also very perceptible, both to the eye and touch between second and third left cartilages, where a marked quivering motion (*fremissement*) attends it. The impulse, however, over the prominence beneath the right clavicle greatly exceeds that of the heart. The first sound only, is heard at the left nipple and in the axilla—it is strongly whizzing, sometimes rasping; near the sternum, on the line of the fourth rib, both sounds are heard; *saw sound* exists over the aortic valves. Along the right upper margin of the sternum, between the clavicle and second rib, the first sound alone heard, and is intensely rasping. Over the mitral valve, first sound is whizzing, similar to that heard at the apex.

Respiratory sounds are greatly masked by those of the heart, and aorta—the respiration on the right side is tubal beneath the acromial extremity of the clavicle; feebly bronchial with fine crepitus posteriorly. *Left Chest.*—Respiration in upper fourth very feeble, but vesicular; on lower half posteriorly, fine crepitus. The respiration is difficult, but is not accompanied with stertor.

Arteries throughout the body, with the exception of those of the arms, beat with great force—pulsation of the radial arteries of very small volume, and corded.

Death took place six hours after this note was written.

*Autopsy, thirty hours after death.*

No infiltration into the cellular tissue; slight emaciation.

*Cranium.*—Effusion of blood into the muscles beneath the scalp; very firm adhesion between the dura mater and the scull at the summit of the left hemisphere. The left hemisphere of the brain is smaller than the right, its convolutions being flattened, whilst those of the right are full and convex; the sinuses and blood vessels of the meninges greatly engorged. On the summit of the middle lobe of the left hemisphere a depression of one inch in length, by one-fourth of an inch in breadth and depth, exists in one of the convolutions; the parietes of this cavity are softened, of a straw colour, and the pia mater dipping into it is of a bright arterial redness, its capillaries much distended and innosculate freely. Arachnoid covering left hemisphere thickened, opaline: numerous gray granulations seen along the left margin of the longitudinal fissure, and on the posterior lobe of the cerebrum. Cortical substance pale, not softened on the convex surface, except at the depressed point. At the base, arachnoid thickened, opaque, especially on the left, near the pons varolii, where the cortical substance is slightly softened. Medullary substance firm, not injected; ventricles contain each about ʒij of bloody serum.

*Cerebellum* normal, with exception of thickness and opacity of tunica arachnoidea.

The arteries of the brain are partially filled with dark blood; their tunics are thickened by numerous cartilaginous patches beneath the serous coat.

*Chest.—Heart.* Upon opening the chest, the heart is found to coincide with the percussion during life.

*Pericardium* contains half an ounce of reddish serum; no adhesions with the heart; is generally of dull white appearance, but without any raised patches upon its surface. The heart, more globular than natural, measures, after being partially emptied of blood, twelve inches in circumference. It contains in its cavities firm fibrinous coagula, which are elastic, tinged with blood, and adhere firmly to the muscular tissue. Right auricle and ventricle much dilated,—would contain a goose egg. Walls of ventricle measure in the middle, exclusive of columnæ carneæ, which are hypertrophied, five lines; at the base and apex, three lines. Left ventricle slightly dilated; walls much thickened,—measure in the middle, exclusive of the columnæ, one inch four lines—at the apex six lines—at the base nine lines. Left auricle more dilated than ventricle; muscular tissue of both auricles hypertrophied; parietes of the left, three lines in thickness. Endocardium of left ventricle and auricle thickened, opaque; mitral valves double usual thickness, with cartilaginous patches upon the anterior valve; circumference of the auriculo-ventricular orifice three inches nine lines. Tricuspid valves flexible, transparent, not thickened; circumference of right auriculo-ventricular orifice four inches six lines; the valves do not entirely close the orifice, and would allow regurgitation. Circumference of pulmonary artery at the semilunar valves three and a half inches; valve is very slightly thickened in points, but is perfectly flexible. The aorta at its orifice is three inches in circumference; all of the aortic semilunar valves contain patches of cartilaginous deposit,—that which is nearest the anterior margin of the mitral valve has its cardiac surface much roughened from an ossific deposit in the sinus of Valsalva. Immediately above the valves the aorta becomes contracted by a cartilaginous deposit beneath the serous coat, its circumference at this point being two and a half inches. The artery above this dilates, and at the summit of the arch measures four inches in circumference,—opposite the innominata, the circumference is three inches,—and a little beyond, in the descending aorta, two and three-quarter inches. The internal surface of the artery, rough from ossific and cartilaginous patches. The innominata and left subclavian arteries, are dilated at their orifices, but are partially filled by the fibrinous coagula formed in the dilated sac of the aorta.

*Lungs.*—Strong adhesion of right lung to the ribs; no effusion into chest. Middle and lower lobe much distended, very heavy, containing numerous black masses of variable size, terminating by abrupt margins; these masses are friable, not hepatised, and sink in water. Bronchial tubes dilated, mucous membrane much injected and thickened. Upper lobes of both lungs crepitating on pressure; margins full and rounded from dilated vesicles. Lower lobe of left lung splenitised, and contains a few apoplectic nodules.

*Liver* smaller than usual, very heavy, firm, resisting the knife; large vessels congested; yellow acini much developed; bile abundant in gall bladder, dark colour and tar-like consistence.

*Stomach.*—Mucous coat of stomach dark gray colour, thickened; pyloric orifice diminished by scirrhous induration. Nothing remarkable in the other viscera, except ecchymosis on mucous coat of small intestines.

*Remarks.*

The point of the greatest interest in this case, is the connection of the cardiac disease with the repeated apoplectic attacks. In the first instance, all the symptoms were very evanescent, lasting but a few hours; thus proving that the suspension of motion and consciousness must have been the result of congestion of the blood vessels of the brain, unattended by any effusion upon that organ. The second attack was of far more serious character—the paralysis continuing after very energetic treatment had been adopted. Gradually, sensation is restored, improvement in motion is manifested, but before the entire use of the limbs is regained, a third attack of paralysis takes place, and death ensues. Disease of the heart had preceded the apoplectic tendency, and had increased until unequivocal signs of hypertrophy, valvular disease, and aneurism of the arch of the aorta were manifested.

The character of the cerebral lesion is interesting. Notwithstanding the perfect hemiplegia, no effusion of blood could be detected in the brain. A small depression on the summit of the left hemisphere, indicated that an effusion had existed at a previous period, and the flattening of the convolutions may be attributed to a similar cause. But, since no effusion existed to explain the cause of the last paralysis, to what can it be imputed? It was observed some days previous to death, that the pulsations at the wrists were comparatively feeble, whilst the carotid arteries beat with great force; the cause of the phenomenon was explained, upon making the post mortem examination, by the presence of coagula, at the orifices of both subclavian arteries, by which, in a great measure, the arterial blood was prevented reaching the arteries of the arm, and was driven with great force to those of the brain. This organ, we have reason to believe, had not entirely recovered from the lesion occasioned by a recent attack of apoplexy—the convolutions of the left hemisphere were depressed by the effects of the hæmorrhagic effusion. Under these circumstances, might not the violent action of the heart, by forcing an anormal quantity of blood into the brain, greatly distend the arteries, engorge the veins and sinuses, and thus produce upon the hemisphere, atrophied by previous disease, all the effects of apoplectic hæmorrhage ?

The whizzing sound accompanying the systole of the heart, and which was heard over the left portion of the præcordial region, seems to have depended upon regurgitation of blood through the mitral valve, inasmuch as it was always heard loudest over that valve, varying under circumstances when the circulation became irregular, whilst no corresponding change was observed over the aortic valves.

Over the dilated aorta, the first sound only was heard, although both sounds existed at the aortic valves.

